# Impact of Dry Chemical-Free Mechanical Pressing on Deagglomeration of Submicron-Sized Boron Carbide Particles

**DOI:** 10.3390/nano15080611

**Published:** 2025-04-16

**Authors:** Mahmoud Elkady, Timo Sörgel

**Affiliations:** Center for Electrochemical Surface Technology ZEO, Aalen University of Applied Sciences, Beethovenstr. 1, 73430 Aalen, Germany

**Keywords:** boron carbide, submicron particles, pressing technique, deagglomeration, chemical-free

## Abstract

Submicron particles are widely used in industrial applications due to their unique physical and mechanical properties that enhance the performance of composite materials. In particular, boron carbide particles are valued for their exceptional hardness and high wear resistance and are especially valuable in protective coatings and aerospace applications. However, these particles can agglomerate, significantly impairing their effectiveness. When this occurs during the development of composite materials, physical and mechanical properties are negatively affected. In this paper, a chemical-free method using a non-destructive, open-system dry mechanical deagglomeration technique is developed, leaving the primary particles unaltered, while breaking up strong adhesions between primary particles resulting from the manufacturing process. This method was tested for the deagglomeration of as-received boron carbide submicron particles, with an average primary particle diameter of d_50_ = 300 nm, and its effect on particle size distribution is presented. Furthermore, X-ray diffraction and true density measurements were carried out on the raw powder. Submicron particles in the dry and as-received state were poured into an experimental mold without a dispersing agent or a protective atmosphere. Static pressure was applied up to 141 MPa to produce tablets at room temperature, finding that 70 MPa yielded the best results in terms of homogeneity, dispersibility, and reproducibility. In order to break apart the densified pressed tablets, ultrasonication was applied before running particle size measurements in the wet dispersed state. Using a tri-laser diffraction light scattering technique, it was determined that particle size distribution followed a Gaussian curve, indicating that this method is suitable to regain the primary submicron particles with uniform properties. It is also shown that applying ultrasound on the as-received powder alone failed to cause the complete deagglomeration of strongly adhering primary particles. These findings suggest that there is no significant wear on the primary particles and no alteration of their surface chemistry, due to the lack of any chemically supported mechanisms such as the alteration of surface charge or the adsorption of surfactants. Furthermore, as the static pressure exerts an immediate impact on all particles in the mold, there is a clear economical advantage in terms of a shorter processing time over other deagglomeration methods such as high shear mixing.

## 1. Introduction

Tribological properties are a major focus of many engineering applications, including aerospace [1,2], aircraft [3,4], automotive [5,6], mining [7], wagon engineering [8] and marine sectors [9]. These specific physical and mechanical requirements often exceed the capabilities of single-phase materials. Composite materials are a promising alternative to traditional materials due to their unique and extended range of low friction and high wear resistance in various tribosystems [10,11,12,13,14] and mechanical properties, like microhardness [15,16,17]. They are created by dispersing one or more solid phases in another solid matrix, e.g., by means of composite electroplating [15,18,19]. Dispersoids usually take the form of fibers, spheres, flakes and platelets. The use of materials based on conventional micrometer-sized particles leads to a limitation of the achievable physical and mechanical properties, sometimes conflicting with the requirements. The demand for specific mechanical properties requires a homogeneous distribution of non-agglomerated particles into the matrix. In the very first step, this requires agglomerate-free starting materials. Agglomeration itself is mainly a phenomenon observed in submicrometer particles, as their total energy by volume (bulk plus surface contributions) is higher than that of larger particles in the micrometer range, due to a much larger surface area. In agglomerates, primary particles are often held together mainly by van der Waals forces, as well as by mechanical interlocking due to shape-related entanglement [20,21]. These forces are relatively weak compared to chemical bonds, making the agglomerates susceptible to breakage into primary particles under shear force [22,23], without altering the latter. This reduces the quality of submicron powders and, thus, the properties of the end product, unless controlled deagglomeration occurs before further processing [24,25]. Nevertheless, to achieve certain properties of superhydrophobicity [26], or to find an alternative to conventional hard chromium electrodeposited layers [27], the use of reinforcing particles is a prospective option.

Boron carbide (B_4_C) particles are ideal for applications in extreme environments [28]. This ceramic is the third overall hardest material known (HV_100_ = 47 GPa) [29,30], with a high elastic modulus (≈450 GPa) [31], an excellent wear resistance [32], an exceptional chemical resistance [33], a high melting point of 2450 °C [34,35], and a low bulk density of 2.52 g/cm^3^ [34,35]. Because of these properties, B_4_C is a promising potential candidate for the development of advanced composites using composite metals [36,37,38,39], polymers [40,41,42,43] and ceramic [44,45,46] materials, additive manufacturing [47,48,49], powder metallurgy [50] and electrochemical/electroless composite layers [28,51,52,53,54,55,56,57,58]. When well-dispersed in a matrix, B_4_C nanoparticles not only enhance mechanical properties [55], but also increase the wear resistance [28,52,53,56,59,60,61] and hardness [51,52,54,56] of the composite. For example, the addition of B_4_C to epoxy resin up to 2 wt.% improves ballistic properties and enhances the matrix toughening and impact energy of the studied composite [43]. The finely tuned microstructure of the matrix and the homogenous dispersion of the B_4_C particles are credited with improved tensile strength [37], hardness [33], and compression strength [62], measured at AA-6061 and AA-2024, respectively. Introducing 1.5 wt.% of B_4_C (particle size ≈ 68 nm) increases Vickers hardness and tensile strength properties of Al-7150 matrix by approximately 22% and 90%, respectively, in comparison to the raw state of the Al-7150 alloy [38]. Particles of boron carbide with an average size of 1.5 µm were used as a reinforcement (without any additional treatment) for codeposition with electroless nickel (Ni-P) on the cast magnesium alloy AZ91D as a substrate for increasing wear and corrosion resistance [59].

These reinforcements can even be submicron-sized. Integrating even smaller submicron particles (particle size ≤ 1 µm) [52] would enable the improvement of the abrasion resistance of composite materials. However, reinforcements of this size are difficult to incorporate into the matrix, as submicron particles tend to agglomerate during processing [24,25].

Conventional methods for preventing reagglomeration and deagglomerating of particles include chemical [34,35] and mechanical approaches [63,64,65,66], respectively. The chosen pH value depends on the point of zero charge (PZC) [67] and can significantly affect properties, such as the colloidal stability, microstructure, and rheological nature of suspensions containing cellulose nanocrystals [68] and goethite nanopowder [69]. Chemical surfactants used to prevent reagglomeration of nanoparticles include cetyltrimethylammonium bromide (CTAB) [55,63,70], sodium dodecyl sulfate (SDS) [51,53,60,63,71,72,73,74], polyethylene glycol (PEG) [63,75,76,77], polyvinyl pyrrolidone (PVP) [23], hexadecyltrimethylammonium chloride (HTMAC) and dodecyl dimethyl benzyl ammonium chloride (DDBAC) [76], trileucine [78], and ammonium salt of polyacrylic acid [79]. However, chemical methods come with a major drawback, namely that the chemical additives can have detrimental effects on the composite production process.

Concerning further potential techniques, a lump breaker was utilized to crush an asphalt pavement into particles with d = 50 mm [80] and a powder crusher was used for grinding W and Zr particles [81]. In order to reduce coal size, different devices such as rotary beakers, roll crushers, hammer mills, impactors, tumbling mills, and roller mills were screened and reviewed [82]. The operational speed of the mentioned devices could be decreased down to 18 rpm, while maintaining a high enough power to reduce the particle size. The literature does not report on the deagglomeration of particles using one of these devices. As the main goal here is to deagglomerate as-received boron carbide into the primary particles without wearing the latter off, these methods were excluded from the current research.

To achieve the effective deagglomeration of nanoparticles mechanically, a number of instruments and methods have been developed, including ball milling, high-pressure homogenization, ultrasonication, and high-shear mixers. These are shown in Table 1.

The literature reports that sonication and centrifugation after cryo-milling procedures help deagglomerate two-dimensional h-BN nanopowder [66]. B_4_C dispersion has been achieved through chemical treatment followed by ball milling for 24 h [34,35]. Mechanical methods, such as ball milling, are easily available and inexpensive for deagglomerating particles, but they can be time consuming. Although the process of particle deagglomeration using ball milling is generally clean, it can produce fine particles that need to be controlled to minimize the environmental impact. The use of a high-shear mixer device enables the rapid deagglomeration of nanoparticle aggregates, resulting in a uniform and stable nanoparticle suspension; however, this technique is typically more cost-effective when applied to large-scale production [83].

In order to deagglomerate silica particles by shear stress, the study from Gerde et al. [84] applied dispersion to a suitable aerosol through the use of a high-pressure nozzle in the range of (1 MPa to 8 MPa). Such a pressure resulted in particle damage, followed by a significant reduction in particle size. This method of particle deagglomeration through the use of a high-pressure nozzle has been used in a number of studies with different pressures [21,78,85,86,87,88] or pressure with shock waves [89,90,91,92]. Heat treatment using the sparking plasma sintering method was used to deagglomerate raw detonation nanodiamond powder with d_50_ = 203 nm [76]. The powder was placed in a graphite mold with a diameter of 20 mm by using a heating temperature of 500 °C, a unidirectional pressure of 30 MPa (t = 5 min), and an argon gas protective atmosphere.

**Table 1:** Conventional particle deagglomeration techniques.

Treatment Technique	Main Operational Principle	Applied Particle Primary Size/ Particle Size Distribution Method	Advantages	Disadvantages
Ultrasonic homogenizer	A probe emits ultrasonic waves into a liquid sample, creating cavitation for dispersion.	Porous SiO_2_ (d = 15–20 nm)/dynamic light scattering (DLS), employing a He–Ne laser [93].	Fast, efficient, reliable, and capable of processing samples in volumes ranging from a few millilitres to several litres [94].	Generates a large amount of heat, it suffers from high energy consumption, scalability issues [94], and reagglomeration [95].
		Al_2_O_3_ (d = 50 nm)/elemental mapping of scanning electron microscope (SEM) [96].		
		Boron nitride nanotubes with a diameter ranging from 5 nm to 10 nm/SEM, transmission electron microscope (TEM) [97].		
Ultrasonic bath	Sample placed in a liquid bath; ultrasonic waves propagate uniformly through the medium.	TiB_2_ (d = 100–200 nm)/laser diffraction [98].	It does not require physical contact with the particles, reducing contamination risks.	Some energy is lost as heat, reducing the overall efficiency compared to direct ultrasonic probes; causes reagglomeration [95].
Hydrostatic pressure + ultrasonic treatment	Ultrasonic treatment using sonotrode under a hydrostatic pressure.	Multi-walled carbon nanotubes (MWCNT Ø 5 nm to 12 nm, L ≈ 50 µm)/DLS, SEM, and TEM [23].	Non-thermal, retains material properties.	Requires specialized, high-pressure systems.
		SiO_2_ (d = 12 nm)/polarization intensity differential scattering (PIDS) [99].		
Ultrasonic jet dispersion	Water supply at a pressure of 400 MPa with nanosuspension. High-speed jet combined with ultrasonication directly impacts particle suspensions.	- AlO(OH) boehmite (d = 0.1–0.8 nm) - Nanodiamond (d = 4–6 nm) - MWCNT (Ø 10–20 nm, L > 2 μm)/laser diffraction [100].	High-speed jet combined with ultrasonication directly impacts particle suspensions.	Requires specialized equipment and it is energy intensive.
RESS/REHPS *	High shear stress in the nozzle passing through the Mach disc in the freely expanding jet.	Al_2_O_3_ and TiO_2_ (d = 1–3 µm)/scanning mobility particle spectrometer, aerodynamic particle sizer, and SEM [21].	Produces fine particles with no solvent residue.	High cost, limited scalability.
Shock waves	Shock-tube filled with argon. Agglomerates suspended in a gas phase by means of shock waves, pressure, and shear forces.	SiO_2_ (d = 40 nm), SiC (d = 1.5 µm)/laser particle size analyzer (Mie scattering principle) [91].	Effective for hard agglomerates, non-contact method.	Equipment complexity, high energy consumption.
High-shear mixer	A rotor–stator assembly creates a high-speed shearing action in a suspension with particles.	SiO_2_ (d = 12 nm)/laser particle size analyzer (Mie scattering principle) [83].	Suitable for low-/high-viscosity materials, scalable.	Can damage sensitive materials
+ ultrasonication		TiO_2_ (d ≈ 25 µm)/laser diffraction and PIDS [22].		+ potential thermal issues.
Swirl airflow	Powder packing in a capsule, discharging to the vortex chamber for dispersion and deagglomeration through swirl airflow-induced capsule rotation.	Drug powders (d = 60–140 µm)/ numerical simulation [101].	Simple design, low energy Requirements.	Limited control over fine particle sizes.
Planetary centrifugal force	Dual-axis mixing generates centrifugal and shearing forces in a container.	Graphene nanoplatelets with 8–15 nm thickness/digital optical microscope [102].	Uniform mixing, suitable for viscous materials.	Limited throughput, high maintenance costs.
Ball milling	A container contains grinding balls and suspension roll on a roller–mixer. The high shear rate breaks particle clusters mechanically.	UO_2_–PuO_2_ (d = 100 nm)/dry route laser granulometer (Mie diffraction theory) [77].	Versatility, efficiency, and safety for diverse sample processing needs are ensured by their ability to handle multiple samples, prevent cross-contamination, and reduce aerosolization [103].	In a laboratory setting, this type of homogenization is limited in terms of sample size. A single tube will hold only a few grams or millilitres of the sample for processing [103].
		Zero-valent iron (d = 200 nm)/ laser diffraction and SEM [104].		
	Mechanically reducing solid particle size by intense agitation of a slurry of the material being milled and coarse milling media [105].	Onion-like carbon ink (d = 30–300 nm, after treatment)/dynamic light scattering, Raman spectroscopy, TEM [73].		
Rotary kiln	Thermal treatment—a rotating inclined cylinder heats (600 °C) and tumbles powder continuously.	Graphite (d_50_ = 375 µm)/dynamic image analysis and dry sieving analysis [106].	Handles large volumes, effective for heat-tolerant particles.	Energy-intensive, limited to thermally stable materials.

* RESS/REHPS—rapid expansion of supercritical solutions/high-pressure suspensions.

Currently available methods often lead to highly and reproducibly deagglomerated particles. However, these methods are expensive [21,91,93,96,97,100,106] and can process only small particle quantities [21,103]; alternatively, they are low-cost but produce low-quality particles [102,103]. These particles potentially reagglomerate, ultimately resulting in a costly or substandard final product [82,94]. Even mechanical dispersion methods often require the use of chemical additives or heat treatment [76,106] to avoid reagglomeration in the dispersed state. It was particularly challenging to find relevant information in open-source literature on the operational and experimental costs of the methods mentioned in Table 1 for a quantitative comparison with our approach.

In this study, a mechanical method using shear force that is generated inside the press mold at room temperature under atmospheric conditions was developed and tested. This method was applied to disperse primary boron carbide submicron particles with a d_50_ of 300 nm. The method was tested, and a monomodal distribution curve was found after pressing with a holding time of 10 s, without altering the physical shape, wearing off the primary particles, or using chemical methods such as pH alteration or chemical surfactant addition. Furthermore, the static pressure in the mold has an immediate impact on all particles in the mold, a phenomenon that is advantageous over stochastic methods, which only treat a portion of the whole sample at a time. In combination with the comparatively short processing time of just 10 s, which is orders of magnitude shorter than that of alternative methods, such as stochastic shear mixing, a clear economical advantage can be expected.

## 2. Materials and Methods

Commercially available B_4_C particles (IHK 915, Industrie Keramik Hochrhein GmbH, Wutöschingen, Germany) with an announced d_50_ = 300 nm were utilized. The shape of the raw powder is irregular. The chemical composition and physical properties of the B_4_C particles are provided in Table 2 and Table 3, respectively, as stated in the manufacturer’s datasheet.

X-ray diffraction (XRD) measurements were carried out on the raw boron carbide powder using a Seifert diffractometer (Sun XRD 3003, GE Sensing & Inspection GmbH, Alzenau, Germany) with three Meteor1D detectors. The X-ray diffractometer was equipped with a Co-tube (acceleration voltage = 50 kV, current = 5 mA, and Co Kα (λ = 1.78896 Å)) in the range of 2θ = [30°, 140°], at a step width of 0.02° and a step time of t = 30 s. True density for the raw powder was measured using gas pycnometer (Ultrapyc 5000 Micro, Anton Paar, FL, USA). The powder, with a volume of 0.5527 cm^3^, was placed into the test cylinder (volume = 4.5 cm^3^). The density measurements were performed across 15 runs at 20 °C in an argon-protective atmosphere with a supported pressure of 0.896 bar for 1 h 24 min.

To achieve mechanical deagglomeration using the method suggested here, and to measure the effectiveness of the particle deagglomeration, different forces (Table 4) were applied to the lower punch of the press mold filled with B_4_C particles using an upstroke hydraulic press (TP 400, Fontijne Holland BV, Vlaardingen, The Netherlands). The upstroke hydraulic press has a plate size of 320 mm × 320 mm, a hydraulic pressure of 200 bar, and a maximum pressure force of 400 kN. Forces ranging from 10 kN to 100 kN were applied, and these values were then converted into pressure values in megapascal (MPa) based on the diameter of the experimental press mold.

**Figure 1:**
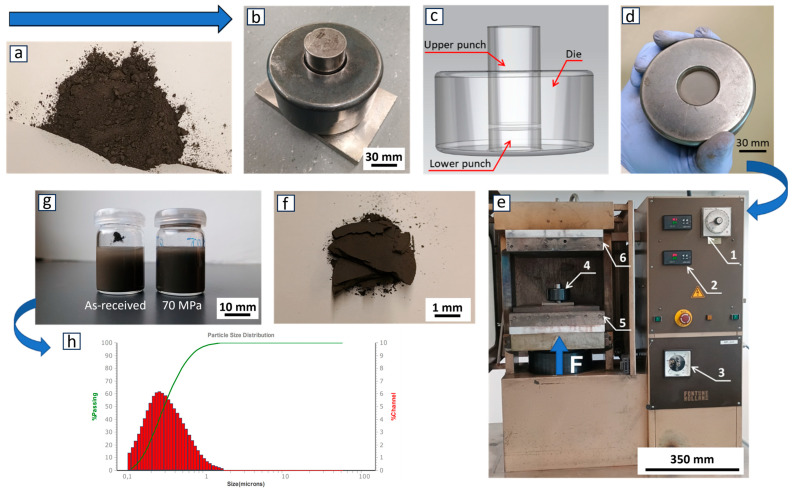
Schematic diagram of the process of dispersion and analysis of B_4_C submicron particles. (**a**) As-received particles, (**b**) photograph of the press mold, (**c**) 3D CAD render of the press mold, (**d**) powder in the mold after pressing, (**e**) hydraulic press—1. holding time control, 2. temperature control, 3. force control, 4. press mold, 5. lower plate, 6. upper plate, (**f**) pressed powder after release from the press mold, (**g**) ultrasonicated powders in deionized water (8 min), and (**h**) particle size distribution after the application of 70 MPa and ultrasonics (8 min + 1 min).

Figure 1 describes the various steps in the deagglomeration method for the boron carbide particles. The raw particles (Figure 1a), with a weight of 3 g, were poured into the experimental press mold (Figure 1b,c), with an internal diameter of 30 mm. Then, the forces presented in Table 4 were each applied to 10 sample groups (groups no. 4 through no. 13). Figure 1d shows the pressed particles in the press mold after applying a defined force for a holding time of 10 s. To investigate the influence of the applied force on the particles’ deagglomeration, three sample groups were used: the as-received sample (group no. 1) and the ultrasonicated sample without the application of pressure (groups no. 2 and no. 3). The experiments were performed in an open system in an upstroke hydraulic press (Figure 1e) at room temperature without the support of a protective gas. After mechanical dry pressing, the particles formed a tablet (Figure 1f) with a thickness of 0.8 mm. A part of this pressed-powder tablet (average weight of approximately 0.015 g) was transferred to a closed glass bottle, which was filled with 4 mL of deionized water. The dispersion was shaken well by hand before treatment in an ultrasonic bath (Figure 1g). The ultrasonic bath, containing a total volume of water of 5 L, was applied at 35 kHz (DT 514, Bandelin Sonorex, Berlin, Germany) to the experimental particles for 8 min at room temperature. It should be mentioned that this ultrasonic treatment was solely applied to break apart the pressed tablet, which was produced by the mechanical deagglomeration step itself, already containing solely primary particles, held together by the applied pressure, but showing no strong interparticle adhesion, as is typical for the agglomerates in the as-received powder. The particle size distribution is one of the most important parameters to evaluate particle dispersion quality. Therefore, particle size in deionized water was estimated using a tri-laser diffraction light scattering analyzer (S3500, Microtrac Retsch GmbH, Haan, Germany). This analyzer can detect particle sizes in the range of 0.02 µm to 2800 µm in a suspension. It was used in order to measure the particle size distribution after each mechanical deagglomeration and in the as-received reference state (Figure 1h). The duration of the applied built-in ultrasonics inside the laser diffraction analyzer was 1 min in the offline mode before performing particle size measurements. The total duration of the ultrasonic treatment before performing the measurements was 9 min. The laser diffraction analyzer supports three red lasers and its algorithms utilize Mie compensation and modified Mie calculations for the non-spherical particle wet method [107]. The measurements were reproduced three times for each sample. Data analyses adhered to DIN [108,109] standards for analyzing multimodal distributions.

## 3. Results and Discussion

**Figure 2:**
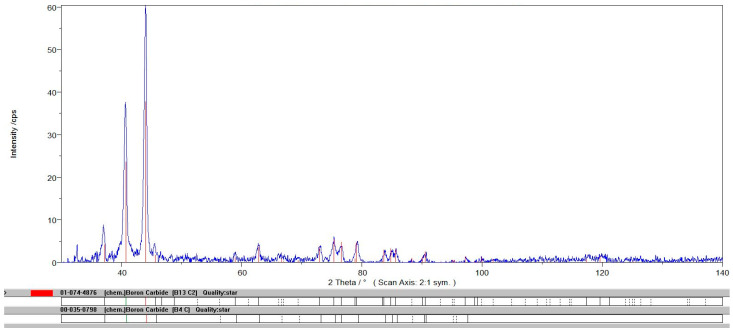
XRD measurements for raw powder of boron carbide.

The XRD measurement (Figure 2) of the industrial B_4_C powder demonstrates additional peak positions of the boron-rich boron carbide phase B_13_C_2_ [110]. Shen et al. [111] observed that the B_13_C_2_ has a lower critical shear strength of 28.6 GPa compared to that of B_4_C, i.e., 46.7 GPa. However, B_13_C_2_ demonstrates a larger critical strain for amorphization, indicating that boron enrichment helps to reduce the amorphization in boron carbide. The elastic modulus of boron carbide material composition manufactured by chemical vapor deposition exhibited the maximum value: E = 475 GPa for B_13_C_2_ [112,113].

The B_4_C raw powder underwent true density measurements and demonstrated an average equal to 2.44 ± 0.02 g/cm^3^. The literature states that the bulk density of boron carbide increases linearly with carbon content within the homogeneity range of the phase and mentions that B_13_C_2_ density is 2.488 g/cm^3^ [112,114,115], in comparison to pure B_4_C which shows a density of 2.52 g/cm^3^ [112,114,115]. It should be mentioned that there is a reasonable difference in results between true and bulk density measurement methods. The bulk density encompasses the surface pores and internal voids of the volume [116], but true density excludes all voids or spaces within the volume, such as pores or gaps [117]. These results suggest that the investigated industrial boron carbide powder has a boron content of at least 78.3 wt.% [112,114,115].

**Figure 3:**
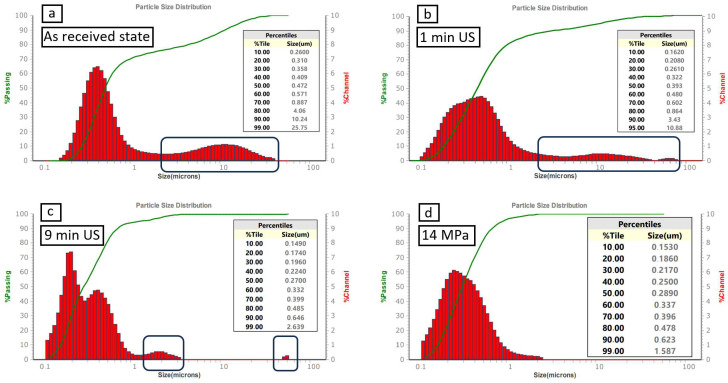

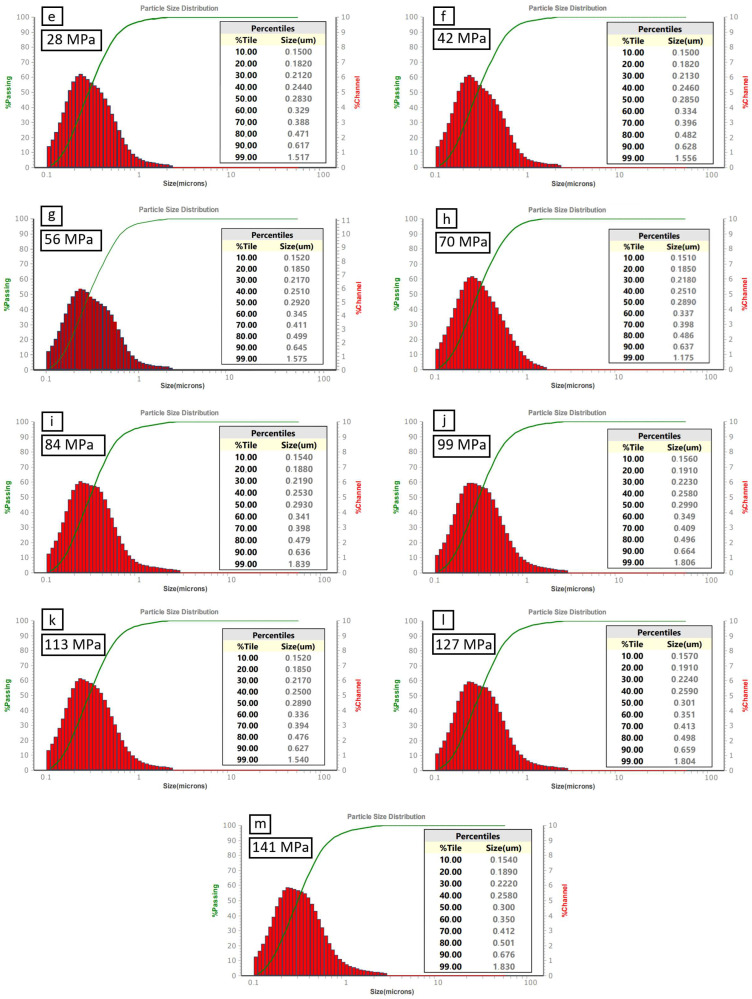
Quantitative distributions of the size of boron carbide particles ultrasonicated and dispersed in deionized water: (**a**) as-received state, (**b**) after 1 min of ultrasonication, (**c**) after 8 min + 1 min of ultrasonication, after an applied pressure of (**d**) 14 MPa, (**e**) 28 MPa, (**f**) 42 MPa, (**g**) 56 MPa, (**h**) 70 MPa, (**i**) 84 MPa, (**j**) 99 MPa, (**k**) 113 MPa, (**l**) 127 MPa, and (**m**) 141 MPa.

In the current study, the particle size distribution was analyzed using the laser diffraction method, which enabled us to evaluate the monomodal Gaussian nature of the average values of reproducible resulting curves (Figure 3). It is worth mentioning that the scale is logarithmic. The powder specific surface area indicates a range of 22–23 M^2^/cc, as stated in [118]. A comparison was made of different forces applied to assess their influence on the particle size distribution.

The first three experimental groups (no. 1, no. 2, and no. 3) are shown in Figure 3a (as-received state), Figure 3b (ultrasonicated for 1 min), and Figure 3c (ultrasonicated for 9 min), respectively. The particle size distribution was significantly skewed, indicating a high degree of heterogeneity in particle size. This skew could be due to the presence of agglomerates (marked in black) in the raw powder, which could not be broken up fully via ultrasonication. Effective sonication requires high mechanical energy to overcome van der Waals forces, with dispersion limited by factors like amplitude, energy, duration, and solvent properties [119]. The lack of applied pressure demonstrated a high level of agglomeration, as evidenced by the broad and irregular curve obtained from the laser diffraction analysis.

Experimental groups from no. 4 through no. 13 (from Figure 3d until Figure 3m) were subjected to different pressures, which, upon exceeding a certain threshold, led to monomodal particle distribution curves. The analysis reveals a narrowed, more Gaussian-like, uniform particle distribution curve for the processed powders compared to the raw one. Although the particle size distributions still exhibited some degree of symmetry, deviations from the ideal Gaussian shape were observed. These deviations could be attributed to agglomeration of boron carbide particles. Despite these deviations, the distributions retained some Gaussian characteristics, suggesting that the applied pressure still provides a degree of control over particle size. This indicates that the primary particle volume is distributed symmetrically around the monomodal mean total volume of the particles. It is worth mentioning that the ultrasonication step after pressing solely serves to break the loosely pressed primary particles apart, a step that is necessary to measure their size distribution.

**Figure 4:**
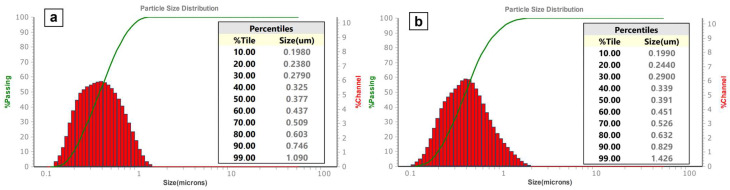
Quantitative distributions of the size of boron carbide particles ultrasonicated and dispersed in deionized water after applying a pressure of 70 MPa to produce (**a**) tablet no. 2 and (**b**) tablet no. 3.

Applying a pressure of 70 MPa (experimental group no. 8) resulted in a monomodal particle distribution curve, with no further improvement in symmetry and shape when further increasing the pressure. A pressure of 70 MPa was chosen as the working point upon which further aspects such as reproducibility (Figure 3h) were investigated. Figure 4 shows particle size distribution curves for the pressed tablets no. 2 (Figure 4a) and no. 3 (Figure 4b). It demonstrates high reproducibility of the suggested dry pressing technique.

**Figure 5:**
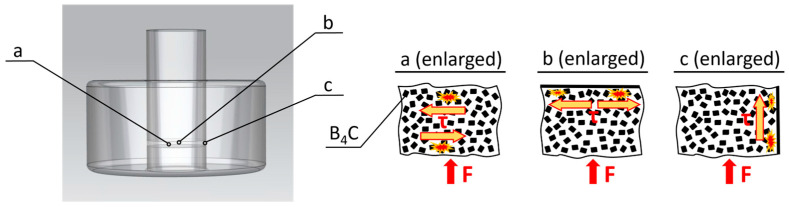
Shear force acting on boron carbide particles during the pressing process.

During the pressing process, boron carbide particles underwent shearing with forces (Figure 5) acting on the interparticle boundaries (Figure 5a) as well as the interfaces between particles and the upper and lower punch, respectively (Figure 5b), and between particles and the mold wall (Figure 5c). These forces contributed positively to the deagglomeration of particles, effectively reducing particle clusters and leading to a more uniform particle size distribution. As a comparison, the literature reports on pressure (30 MPa) applied in a heated press mold (500 °C–1300 °C) to deagglomerate nanodiamond particles followed by ultrasonication [76]. However, the authors did not mention the role of shear force in particle deagglomeration.

The almost static nature of this method leads only to short movements, yet with a high force, so that the method is clearly able to provide the energy needed for deagglomeration without engaging in wear mechanisms, which would generally become more significant with longer relative movements. The latter would be a typical characteristic of, e.g., ball milling, which strongly enhances the risk of primary particle destruction. Another aspect in favor of the suggested method is the fact that particles are mainly interacting with each other, while only a minimal proportion comes into contact with either the mold wall or the punches. In this way, as the inter particle forces typical of agglomerates are still fairly below the strength of the primary particles, this method ideally addresses the described task.

**Figure 6:**
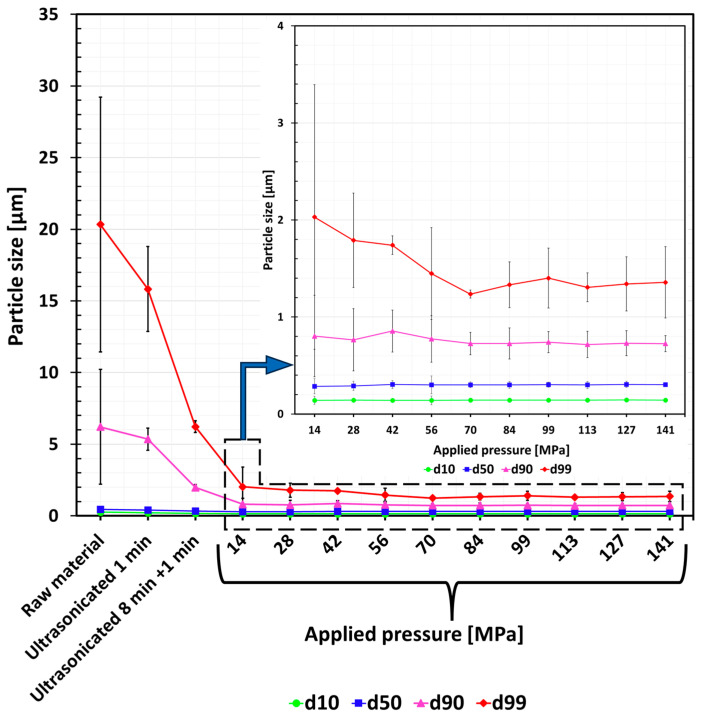
Comparison of boron carbide particle mean size distribution after applying different deagglomeration methods and parameters, including standard deviations for three subsequent measurements.

Figure 6 compares the results of deagglomeration attempts and the differences between ultrasonication and mold pressing with various treatment times and pressures, respectively. While the average curves of three subsequent size measurements are presented in Figure 3, Figure 6 further provides the respective standard deviations. It demonstrates that applying static mechanical pressure resulted in full particle deagglomeration, once a certain threshold was exceeded. Consequently, increasing the force directly translates into a higher degree of particle deagglomeration (group no. 8–70 MPa), confirming the method’s effectiveness and adjustability. All experimental groups have almost the same values of d_10_ and d_50_, with a slight difference in d_90_. Furthermore, the largest differences in the values of d_99_ could be observed before and after applying pressure.

**Figure 7:**
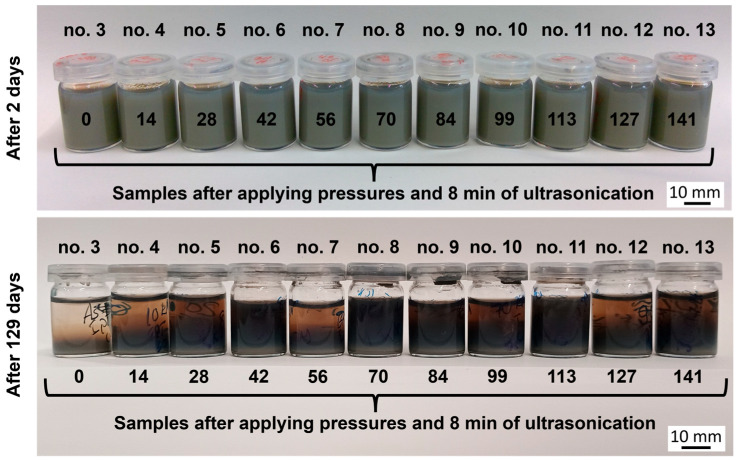
Boron carbide particles sedimented in deionized water after being left for 2 days and 129 days at room temperature, with different pressures and 8 min of ultrasonication subsequently applied.

Figure 7 presents the experimental samples in glasses filled with boron carbide powders with different applied pressures (from 0 MPa through 141 MPa—experimental groups from no. 3 to no. 13, respectively) in deionized water, with the samples subsequently undergoing an ultrasonication process in an ultrasonication bath for 8 min. In semiquantitative sedimentation experiments, a resting period of 129 days compared to 2 days demonstrates only a minor difference in translucence due to particle sedimentation over time for samples that have been subjected to mechanical pressing and ultrasonication (samples no. 6, no. 8 and no. 9). In comparison, sample no. 3, with the raw material solely ultrasonicated (8 min), shows a significant degree of particle settling. Despite the ultrasonication, sample no. 3 obviously still contained unbroken agglomerates which showed a faster settling behavior due to their size. Primary particles of boron carbide have a low driving force to agglomerate in water and even in aqueous solutions with higher ionic strength, as their degree of van der Waals interaction is low. Therefore, the zeta potential developed in deionized water at pH = 7 is fully sufficient to prevent the primary particles from reagglomerating (the point of zero charge of B_4_C is between pH = 6 and pH = 8 [120]). What can be observed from Figure 7 is that, after a rest period of 129 days, all samples which were not fully deagglomerated in the treatment exhibited a faster speed of sedimentation, which led to a faster clearing of the above liquid phase. The explanation for this is that dispersions with the same volume and the same total particle content are best dispersed, in the sense that only primary particles are present, when the sedimented volume is at its lowest. This is because an ensemble of individual primary particles can pack more densely than they could if they were agglomerated.

## 4. Conclusions

An unconventional fast and cost-effective method for deagglomeration of boron carbide submicron particles was developed. It was proven that applying mechanical pressure on boron carbide particles in the dry state results in the deagglomeration of the latter. The particle size followed a monomodal Gaussian distribution curve, suggesting that this method fully restores the primary particles with consistent and uniform properties. Such a distribution is typically desired in materials science, as it ensures the highest degree of reproducibility when further processed. In future work, possible limitations of the presented method need to be identified, e.g., when treating brittle or soft particles which might either alter the primary particle size or lead to plastic deformation. However, to overcome this, these findings provide valuable guidance for optimizing the required pressures to achieve efficient and high-quality particles with minimum agglomeration. Additionally, the economic impact of the developed method has the advantage of rapid execution, which can lead to significant time savings in practical application, highlighting its potential in terms of cost-effectiveness.

## Figures and Tables

**Table 2:** B_4_C submicron particle chemical composition.

Impurities	SiO_2_	Al_2_O_3_	Fe_2_O_3_	CaO	TiO_2_	B_4_C
Percentage	<1.2	<0.1	<0.4	<0.05	<0.1	Base

**Table 3:** Physical properties of B_4_C submicron particles.

Parameter	Value
Theoretical density [g/cm^3^]	2.51
Melting point [°C]	2350
Hardness [HV_10_]	30–40
Thermal conductivity [W/(m·K)]	30
Color	Dark grey

**Table 4:** Applied parameters of the experimental samples.

	Sample Group	1	2	3	4	5	6	7	8	9	10	11	12	13
Parameter														
Applied pressure [MPa]		0	0	0	14	28	42	56	70	84	99	113	127	141
Holding time [s]		-	-	-	10	10	10	10	10	10	10	10	10	10
External US treatment *		-	-	+	+	+	+	+	+	+	+	+	+	+
Internal US treatment **		-	+	+	+	+	+	+	+	+	+	+	+	+

The ultrasonic (US) treatment was applied to the experimental samples in * a separate US instrument for 8 min or ** as part of the laser diffraction particle size measurement procedure (built-in ultrasonication for 1 min immediately before measurement).

## Data Availability

Data is contained within the article.
